# Addressing under-appreciated risk in healthcare simulation

**DOI:** 10.1186/s41077-026-00423-0

**Published:** 2026-03-26

**Authors:** Paul O’Connor, Angela O’Dea, Dara Byrne

**Affiliations:** 1 Department of General Practice, School of Medicine, University of Galway, 1 Distillery Road, Galway, Ireland; 2 Irish Centre for Applied Patient Safety and Simulation, School of Medicine, University of Galway, Galway, Ireland; 3 Health Service Executive, National Simulation Office, Dublin, Ireland

## Abstract

Our experience of running a simulation centre reveals specific areas of risk that are not widely discussed in the simulation literature- namely the potential for physical harm and threats to the viability and continuity of simulation programmes. Physical harm can occur in unanticipated ways and therefore it is imperative that simulationists acknowledge these risks, and mitigate against them, through robust risk management processes. Key operational risks to the viability and continuity of programmes are financial and human resources. The design and delivery of simulation activities requires a team of individuals who complement each other in terms of administrative, simulation and clinical expertise. There is also a need to ensure that the loss of a key member of the team does not threaten the continuity of the delivery of the simulation programmes. Simulation based education (SBE) is an effective approach to the education and training of healthcare providers. However, it is vitally important to be aware of and manage the safety risks of the activities and ensure human resources are available to ensure the viability and continuity of a simulation facility and programmes.

## Introduction

Simulation-based education (SBE) enables healthcare providers to learn and practice in a safe and controlled environment. SBE has positive outcomes and there is strong evidence to support the transfer of learning to clinical practice [1]. Although, as SBE is immersive, complex, and experiential, there are greater associated risks than with traditional didactic teaching methodologies. The risks of psychological harm to learners and the risks associated with in-situ simulation to patient safety are well described in the literature. For example, the risk that simulation interferes with real clinical care [2] or that the clinical environment is contaminated with fake, or out-of-date, medication [3]. However, there are also many other risks of simulation that receive less attention in the simulation literature. For example, the risk to the environment, risks of data and confidentiality breaches, the risks of using artificial intelligence, and the risk of culture bias and reinforcement of health disparities.

The goal of this article is not to provide a comprehensive review of all of the under-appreciated risks associated with healthcare simulation. Rather, we focus on two specific areas of risk we identified during the development of the safety statement and strategic framework for our simulation centre: (i) risks of physical harm; and (ii) risks to the viability of a simulation programme. These risks have the potential to result in consequential- and indeed catastrophic- impacts for patients, learners, simulated participants, and simulation staff. Yet despite the potential impact of these risks, they are under-explored in the simulation literature, and may be under-appreciated by the simulation community. In this paper, we examine these risks within the context of SBE, while acknowledging their relevance to broader applications of simulation beyond education. The purposes of this paper are to:

1. highlight the under-appreciated risks of physical harm to patients, learners, simulated participants (SPs) and simulation staff;
2. highlight risks to programme viability and continuity; and
3. make suggestions for how to mitigate these risks.

## Safety risks to patients, learners, simulated participants, and simulation staff

There are a range of under-appreciated risks to the safety of patients, learners, SPs, faculty and simulation staff.

### Safety risks to patients

Some of the more obvious and predictable risks to patients from in-situ simulation have been acknowledged in the literature. For example, risk of contamination of the clinical environment with simulation only equipment and medicines (e.g. [3, 4]), or the risk to patients from use of clinical resources including staff [2]. Other risks are more subtle but may be just as dangerous. One of these risks is negative transfer. Negative transfer occurs if poor practices carried out in a simulated setting transfer into clinical practice. To illustrate, if poor hygiene practices are not addressed in SBE, then these practices may transfer into the clinical environment. Similarly, an error made by a learner during a simulation that is not addressed in the debriefing, may lead the learner to assume that the action was correct and result in the transfer of this ‘learning’ to the clinical environment where it can cause actual harm to patients [5]. Arguably, an even more insidious risk is that no learning occurs, or the learning fails to transfer to the clinical environment [6]. This risk can be difficult to detect. However, alignment with an appropriate learning theory (or combination of theories) as well as good instructional design and facilitation will help prevent negative learning and will reduce the likelihood of undesirable outcome. Learning theories that are of particular relevance to SBE include: adult learning theory, behavioural learning theory, social cognitive learning theory, and constructivist learning theory [7]. There are many approaches to instructional design. The Analyse, Design, Development, Implement, and Evaluate (ADDIE) model is so commonly applied that it has been described as being virtually synonymous with instructional systems development [8].

### Safety risks to learners

For learners there is the potential for a negative impact of SBE on their psychological and/or physical safety. There is a large literature on creating psychologically safe learning environments (e.g. [9, 10]). However, there are other potential risks of physical harm have received less attention (e.g., the use of clinical equipment such as real defibrillators outside of the controlled clinical setting). These risks can differ from those that exist to healthcare providers performing the same task in the clinical environment. For example, learners may be less vigilant when they are not in the actual clinical environment, or they may falsely assume that the equipment is not real. Just as in the clinical environment, the simulation environment is complex and harm can occur in a myriad of unanticipated ways.

### Safety risks to simulated participants and simulation staff

Due to their close proximity to live training events, SPs may be at risk from inexperienced learners practicing examinations and procedures on them. There is evidence that SPs have had actual procedures performed on them, such as oxygen delivery, venipuncture, and even administration of airway adjuncts [6]. In order to mitigate such harms clear instructions should be communicated to the SP and to the learner about roles, expectations allowed and forbidden actions for all participants in a SP encounter. Simulation staff can also be at risk of physical injury from lifting and carrying or from handling hazardous biological or chemical agents (e.g., cadaveric materials, cleaning agents).

### Mitigating risks to patients, learners, simulation participants, and simulation staff

Mitigating these safety risks requires a robust risk management process with appropriate policies, procedures, governance structures and mechanisms to protect against the physical and psychological risks associated with SBE. Brazil et al. [11] have written a useful overview of the development of a safety policy for a simulation facility. Simulation societies (e.g., Society for Simulation in Healthcare) provide resources and guidance on risk management. It may also be possible to draw upon the health and safety policy, and the support of dedicated health and safety personnel from the parent organisation of which the simulation facility is part.

The approach recommended for mitigating the risk of physical harm is to complete a comprehensive risk assessment for the simulation activities carried out in a facility. Despite the importance of completing a risk assessment, a recent scoping review found a lack of systematic risk assessment processes for simulation activities [12]. Although simulation facilities may operate under the broader policies of their parent organisation it is important that the unique risks present in simulation environments are addressed [12]. There are local contexts and particular activities which are unique to each simulation facility. Every centre will have a specific set of hazards that may create unexpected or unanticipated risks. For example, our centre is attached to a hospital, this creates risks that are not present in a centre that is not on a clinical site. On a rare hot day in Galway, a hospital patient was found in one of the simulation rooms as the doors (which are normally locked shut and accessible via a swipe card) had been propped open to aid airflow [7]. It is crucial that the appropriate stakeholders (such as technicians, faculty, simulated participants, and health and safety representatives) are engaged in the risk assessment process to ensure that all hazards are identified and addressed. It is suggested that risk assessment also has benefits beyond the identification and controls for specific hazards. The process itself can support a positive safety culture by fostering an attitude that managing risk and safety is the responsibility of everyone in the facility and encourages the proactive identification of hazards.

Both the safety policy and the risks assessments should be ‘living’ documents that are actioned and updated on a regular basis. To illustrate, we conduct an annual healthcare professions ‘taster day’ for approximately 200 secondary school children at our simulation centre. Table 1 provides an overview of the hazards and control measures identified for this particular simulation activity. The risk assessment for this activity is reviewed each year before the taster day and it sometimes changes based on the experience of previous years (e.g., cloakroom facilities for coats to prevent students from overheating and so reduce the risk of fainting).

**Table 1:** Hazards and control measures for an health professions ‘taster day’ for secondary school students

HAZARD & RISK DESCRIPTION	CONTROL MEASURES
200 secondary school students will attend the simulation centre on a Saturday (100 students in the morning and 100 in the afternoon). Students will have the chance to observe and actively participate in medical, nursing and health sciences procedures and activities such as simulated birth using a birthing manikin and handle an infant simulator, simulated emergency care of a paediatric patient using a manikin, simulated airway management on a task trainer, manipulating simulated bones and joints, simulated endoscopy, and meet a simulated patient. The potential hazards identified are: • the risk of a student fainting due to standing for prolonged periods, heat of the building or from the realism of the simulated models. • the risk of slips, trips and falls to students and staff. • risk of a large volume of people in the building during an emergency event (fire alarm). • risk associated with out of hours events.	**Risk of fainting** • No needles or sharps are involved in any station. • Limited use of fake blood. • Limited graphic images. • Plenty of student helpers and extra staff available to help unwell students. • Staff that are medically/first aid trained are present. • Medical student helpers briefed to look out for students who look as if they may faint and bring them to the recovery room. • Contact numbers for each student available. • A recovery room for those feeling unwell with cold drinks and snacks. Set up of this room to be assigned to a specific team member. • Sick Bay to have a sign on the door to ensure it is not blocked/used for storage on the day. • Students are warned that they may find the content disturbing and to remove themselves and inform a helper if they feel unwell. • Each group of 10/12 students is assigned a helper to keep an eye on them as they move through the stations • Email sent to attendees in advance of workshop re: fainting, clothing, etc. **Slips trips and falls** • Walk around before event to ensure there are no trip hazards. • Ensure cleaning cupboard is unlocked and mop, bucket, and warning signs are available in case of a wet spill. **Out of hours and large volume of people** • Sufficient staff members who are fire marshal trained to be present. • Security are informed of the event and have it on their calendar. • A sufficient number of members of staff and helpers for every 10–12 students are present. • Students asked to remove their external clothing with cloakroom facilities provided. • On a particularly warm day- windows should be opened and fans set up in rooms with no windows to increase ventilation.

## Risks to the viability and continuity of simulation programme

A simulation facility must be adequately resourced and supported. Key risks to the viability and continuity of simulation programmes include financial and human resources.

### Financial risks

The costs associated with SBE fall into three main categories *‘*stuff, staff, and space’ [13]. The costs of a SBE activity may be underestimated, or even unknown. An understanding of the cost of SBE programmes is necessary to inform decisions about resource allocation, and to ensure that there is sufficient funding available to run planned activities. To illustrate, an Objective Structured Clinical Examination for 185 students at a Scottish medical school was conservatively estimated to cost more than £65,000 [14]. Even if simulation is effective, it does not necessarily mean that the cost of providing it is justified [15]. Therefore, a clear understanding of the cost of delivering an SBE activity provides information about where savings could be made, where there is a need for additional funding, and which SBE activities cannot be supported.

### Human resource risks

SBE requires a team of individuals who complement each other in terms of their administrative, simulation, and clinical expertise. Arguably, the simulation specific expertise is particularly challenging to acquire as much of it may need to be learnt ‘by doing’. For example, simulation technicians require broad range of knowledge (technical, clinical, and pedagogic), skills (resourcefulness, pedagogic, team, and technical), and attitudes (professional and “can-do” mentality) in order to be effective [16]. Many simulation facilities are run by a relatively small number of people. As such, there is a risk to the viability and continuity of programmes should one key individual leave permanently, or for an extended period of time, as it may be difficult to replace their expertise.

### Addressing risks to programme viability and continuity

In agreement with Nestel et al. [17], it is recognised that few people involved in simulation have skills or training in economic evaluation. We have a number of suggestions for addressing risks to a programme’s viability and continuity: (1) engaging with people with expertise in financial management and human resources; (2) strong leadership; and (3) engage in strategic planning.

Simulation programmes need guidance from people with skills in budgeting, procurement, financial management, and human resource management in order to ensure that the strategic and business aspects of the operation is appropriately managed [7]. For many facilities, it will not be possible to have staff with these skills working full-time, or even part-time, in the centre. However, the parent organisation of a simulation centre will likely employ people with these skills. We have found that people with finance skills in our own organisation have been very supportive in helping with tasks such as budgeting and procurement. Therefore, we recommend seeking advice from appropriately skilled people in the wider organisation to provide guidance on financial management of the simulation facility.

Careful oversight is required to ensure that there is sufficient staff, with the necessary knowledge and skill mix, to deliver SBE programmes that can continue- even if key personnel leave. It has been suggested that pressure to accommodate SBE programmes can result in overwork, put a strain on operations, and compromise the well-being of simulationists [18]. Therefore, there is a need for strong, and present leadership at a simulation facility to oversee the demands placed on staff, with sufficient seniority to advocate for junior and to ensure they are supported and not over-tasked. Our parent organisation provides training in human resource skills such as personnel, conflict, and project management. We suggest that those in leadership positions in simulation seek out any human resource training that is offered in the parent organisation. We recommend that there is succession planning to ensure that others are mentored to take on leadership positions in the facility in the future. We also suggest that there is cross-training for staff in the facility to ensure that there are not any tasks that can only be performed by one member of staff (e.g., audio-visual support).

It is important for a simulation facility to have a strategic plan. Strategic planning is critical to the long-term viability and success of a simulation facility [19]. First, strategic planning will support the alignment of simulation activities with the mission, vision and goals of the facility. Second, strategic planning involves identifying the future direction of the simulation facility and the means for achieving the desired future state. This planning will support the identification, and mitigation, of risks to the medium and long-term viability of a simulation facility [20]. For a discussion of the development of a strategy for healthcare simulation see O’Connor et al. [19]. The importance of strategic planning has been identified by international simulation societies (e.g., the Association for Simulated Practice in Healthcare, and the Society for Simulation in Healthcare).

## Conclusion

SBE is widely perceived as safe and effective, yet this assumption can obscure important under-appreciated risks. Addressing these risks requires explicit recognition, robust governance and risk management processes, broad stakeholder engagement, and strategic leadership. By drawing attention to these issues and proposing practical mitigation strategies, we aim to encourage reflection within the simulation community. Sustained attention to both safety and sustainability is essential if SBE is to continue to develop responsibly and realise its potential to enhance healthcare education and patient care.

## Data Availability

Not applicable.
